# Enhanced dispersive solid phase extraction assisted by cloud point strategy prior to fluorometric determination of anti-hepatitis C drug velpatasvir in pharmaceutical tablets and body fluids†

**DOI:** 10.1039/c7ra13719b

**Published:** 2018-04-10

**Authors:** Mohamed M. El-Wekil, Hassan Refat H. Ali, Adel A. Marzouk, Ramadan Ali

**Affiliations:** Department of Pharmaceutical Analytical Chemistry, Faculty of Pharmacy, Assiut University Assiut Egypt mohamed.mohamoud@ymail.com; Department of Pharmaceutical Chemistry, Faculty of Pharmacy, Al Azhar University Assiut Egypt; Department of Pharmaceutical Analytical Chemistry, Faculty of Pharmacy, Al Azhar University Assiut Egypt

## Abstract

An innovative spectrofluorometric method was developed for the analysis of a recently FDA approved anti-hepatitis C velpatasvir (VELP). The developed method was relied on dispersive solid phase extraction (dSPE) using synergistic effect of reduced graphene oxide (RGO) and cobalt hydroxide nanoparticles (CHNPs) in addition to cloud point extraction (CPE) using polyethylene glycol 6000 (PEG 6000) as non-ionic surfactant. This method combines the merits of preconcentration and interferences elimination achieved by dSPE and CPE, respectively. All relevant parameters such as surfactant concentration, ionic strength, pH, incubation time and others were thoroughly investigated and optimized. Fluorometric detection of VELP was carried out at excitation wavelength of 350 nm and emission wavelength of 415 nm. Under the optimum conditions, a linear calibration curve was achieved in the range of 0.5–45 ng mL^−1^. Limits of detection (LOD) and quantification (LOQ) based on three and ten times the standard deviation of the blank were 0.040 and 0.112 ng mL^−1^, respectively. This method was successfully applied for determination of VELP in real samples such as tablets, human plasma and urine samples with good recoveries.

## Abstract 2

Highly sensitive and selective solid phase extraction method combined with cloud point extraction strategy was developed for analysis of velpatasvir in its pharmaceutical tablets and biological fluids.

## Graphical abstract

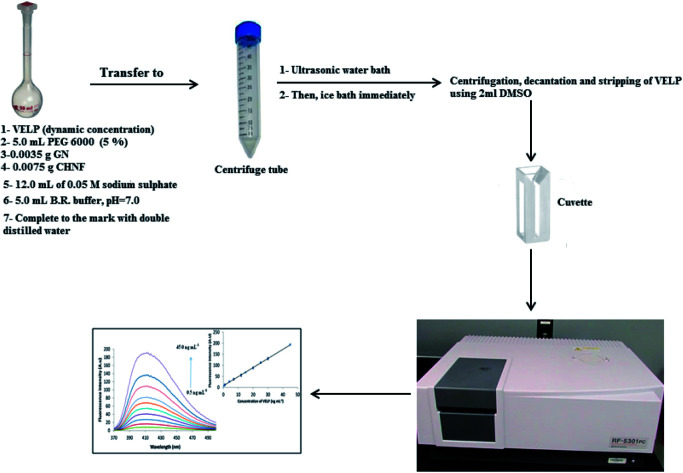

## 1. Introduction

**Fig. 1:**
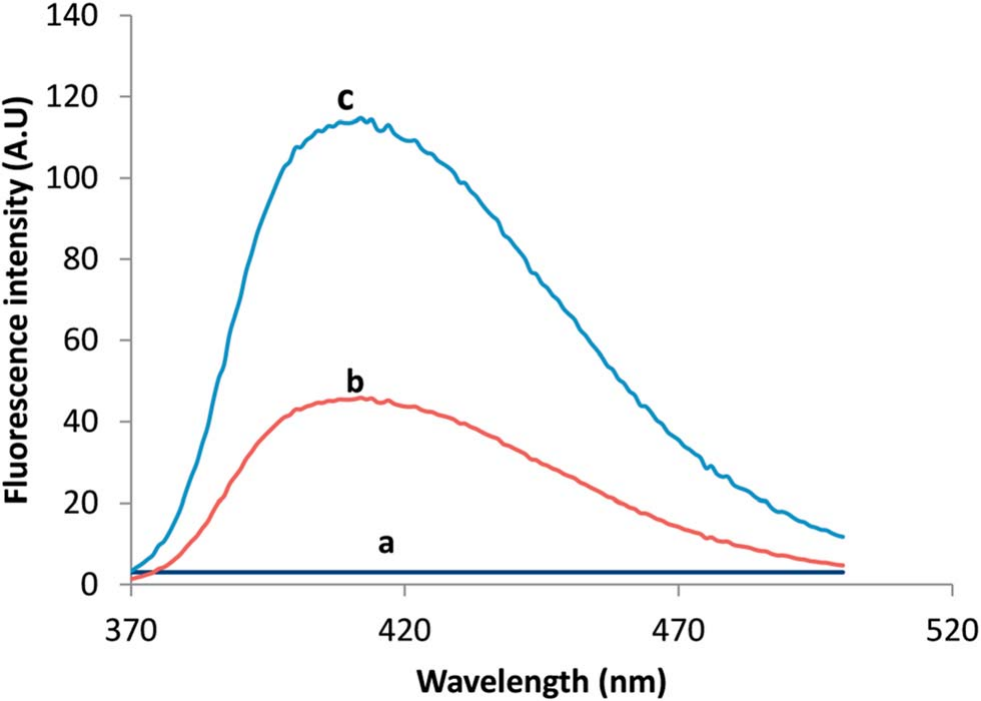
The emission spectra of Blank (a), 30 ng mL^−1^ VELP after RGO/CPE (b) and (c) 30 ng mL^−1^ VELP after RGO/CHNF/CPE.

Hepatitis C virus infection is a major health problem worldwide and no vaccine has been developed against this virus. Velpatasvir (VELP) (Fig. 1S†) is a non-structural protein 5A inhibitors belongs to a class of antiviral drugs called protease inhibitors and usually administered with sofosbuvir for treatment of hepatitis C infection of all six major genotypes.^1–3^ The main merits of VELP are decreasing dose to inhibit HCV replication; once-daily dosing; resistance profiles that do not overlap with those of other direct acting anti-hepatitis C and successful suppression of HCV replication with an acceptable safety profile in early clinical trials.^4^ The *C*_max_ and *T*_max_ of VELP are 259 ng mL^−1^ and 3 hours, respectively after oral administration. Its liver metabolism is very slowly and present unchanged in blood at level exceeds 99% with biological half-life = 15 hours.^5,6^ Previously, anti-HCV drugs were known to cause serious side-effects such as depression and anemia so *in vivo* monitoring is important to prevent the incidence of health disorders.

To the best of our knowledge, HPLC methods were solely developed for analysis of VELP and sofosbuvir in binary mixture.^7–9^ These methods have several disadvantages like time-consuming, large solvents consumption, complicated experimental procedures and low sensitivity if compared with the newly developed spectrofluorometric method.

Many extraction methods to enrich the target analytes from complexed matrices were introduced. These methods include hot continuous extraction (HCE), microwave assisted extraction (MAE), ultrasound-assisted extraction (UAE) and solvent–solvent extraction (SSE). The main drawbacks of HCE, MAE, UAS and SSE are using toxic organic solvents, needing thermally stable compounds, formation of unpredictable free radicals and consuming high amount of toxic solvents, respectively.^10^

Reduced graphene oxide (RGO) is a good candidate used extensively for developing a good adsorption site due to its large surface area, chemical stability and low manufacturing cost.^11^

Dispersive solid phase extraction (dSPE) has many advantages such as rapidity and the provision of more concentrated extracts.^12^ In addition, cloud point extraction (CPE) offers several merits such as low toxicity, high efficiency and simplicity^13–15^ and so finds wide applicability in analysis of hydrophobic analytes especially with interfering substances.^16^

Combination of dSPE and CPE were previously reported for the analysis of Fe(iii) and Cu(ii),^13^ doxazosin and alfuzosin^17^ and arsenic in natural water.^18^

So, the aim of the current work is to develop ultrasensitive, selective and cost-effective spectrofluorometric method for determination of VELP in tablets and biological fluids for the first time. The method simply based on RGO/CHNPs as dispersive solid phase extraction (dSPE) platform and a cloud point extraction (CPE) with PEG 6000 as a nonionic surfactant. The method was successfully applied for the determination of the cited drug in presence of other common co-administered drugs such as sofosbuvir, ribavirin and proton pump inhibitors.

## 2. Experimental

### 2.1. Chemicals

Velpatasvir, sofosbuvir, omeprazole, esomeprazole and ribavirin were kindly supplied from National Organization for Drug Control and Research (NODCAR), Cairo, Egypt. Cobalt nitrate, graphite, methanol and hydrazine hydrate were purchased from Sigma Aldrich. Boric acid, phosphoric acid, acetic acid, sodium sulphate, acetonitrile, dimethylsulphoxide (DMSO), potassium permanganate, sodium nitrate, hydrogen peroxide and sulphuric acid were purchased from El-Nasser for chemicals, Cairo, Egypt. Epclusa® tablets were obtained from the local market. Double distilled water was used in whole study.

Britton–Robinson buffers were made by mixing equal volumes of 0.04M phosphoric acid, 0.04 M boric acid, 0.04 M acetic acid and adjusting pH by 0.3 N NaOH.

### 2.2. Instrumentation

A Shimadzu RF-5301 PC spectrofluorophotometer (Tokyo, Japan) was used for fluorimetric measurements. The slit width of both excitation and emission monochromator was set at 5 nm. The pH values of solutions were measured using Hanna pH meter (Hanna Instruments Brazil, São Paulo, Brazil) with a combined electrode. The solutions were sonicated using ultrasonic cleaner, Branson Transonics Corporation, Eagle Road, Danbury, USA. Surface morphology studies were carried out using scanning electron microscope (SEM), JEOL JSM-5400 LV instrument (Oxford, USA), A Nicolet 6700 FTIR Advanced Gold Spectrometer, supported with OMNIC 8 software (Thermo Electron Scientific instruments Corp., Madison, WI USA) was used to record FTIR spectra and Elemental analysis was done using OXFORD INA Energy Dispersive X-ray Spectrometer (EDX). Raman spectra were recorded with a Bruker Senterra Raman microscope (Bruker Optics Inc., Germany) with 785 nm excitation, 1200 rulings mm^−1^ holographic grating, and a charge-coupled device detector. The average particle size of the prepared nanoparticles were analyzed by photon correlation spectroscopy using a Zeta sizer 3000 HAS (Malvern, Instruments GmbH, Germany).

### 2.3. Synthesis of reduced graphene oxide (RGO)

Graphite oxide was synthesized from natural graphite by modified Hummers method.^19^ Briefly, 2.0 g of graphite powder and 1.0 g NaNO_3_ were mixed, and then put into 96.0 mL conc. H_2_SO_4_ in an ice bath. Under vigorous stirring, 9.0 g KMnO_4_ was added gradually. The temperature of the mixture was maintained below 20 °C. The ice bath was removed and mixture was stirred in a water bath for 2 h. To the brownish color pasty liquid, 150 mL of double distilled water was added. To maintain temperature below 50 °C double distilled water was added continuously till total volume of double distilled water was 200 mL. To the above mixture, 5 mL of 30% H_2_O_2_ was added and it was observed that the solution color transformed into brilliant yellow along with bubbling. The mixture was stirred for 2 h; it was filtered and washed with 10% HCl aqueous solution, water, and ethanol. The product obtained was dried under vacuum at 60 °C. For the synthesis of RGO, 100 mg of graphite oxide was dispersed in 100 mL of water and sonicated for 1 h. In this step conversion of graphite oxide to graphene oxide (brown dispersion) took place. To the above dispersion 2.0 g of hydrazine hydrate in 5 mL water was added and the mixture was refluxed at 100 °C for 24 h under magnetic stirring. Finally, the mixture was filtered, washed thoroughly with water and dried at 60 °C for 12 h.

### 2.4. Synthesis of Co(OH)_2_ nanoparticles (CHNPs)

Cobalt hydroxide nanoparticles were synthesized according to Kong *et al.*^20^ Briefly, Co(OH)_2_ nanoparticles were prepared by a simple precipitation method. The first step was the dissolving of cobalt nitrate as aqueous solution (0.5 M, 20 mL) in a glass beaker, using a magnetic stir bar. The cobalt nitrate solution was slowly adjusted to pH 9 by addition of 5 wt% NH_3_ H_2_O (25 mL) at a temperature around 15 C. The NH_3_ H_2_O was added dropwise with a constant time interval of 15 seconds. The resulting suspension was stirred at this temperature for an additional 3 h. Then the solid was filtered off using 0.45 μm microporous filtration membrane then washed with double distilled water four times. The obtained cobalt hydroxide nanoparticles product was dried at 100 C for about 30 min.

### 2.5. Preparation of standard solutions

VELP stock solution (0.2 mg mL^−1^) was prepared by dissolving appropriate amount (20 mg) of VELP in 100-volumetric flask and the volume was made to the mark with ethanol. The working standard solutions of different VELP concentrations were prepared by diluting the stock solution with ethanol. The prepared solutions should be protected from light to prevent degradation of VELP.

### 2.6. Analytical procedure

**Fig. 2:**
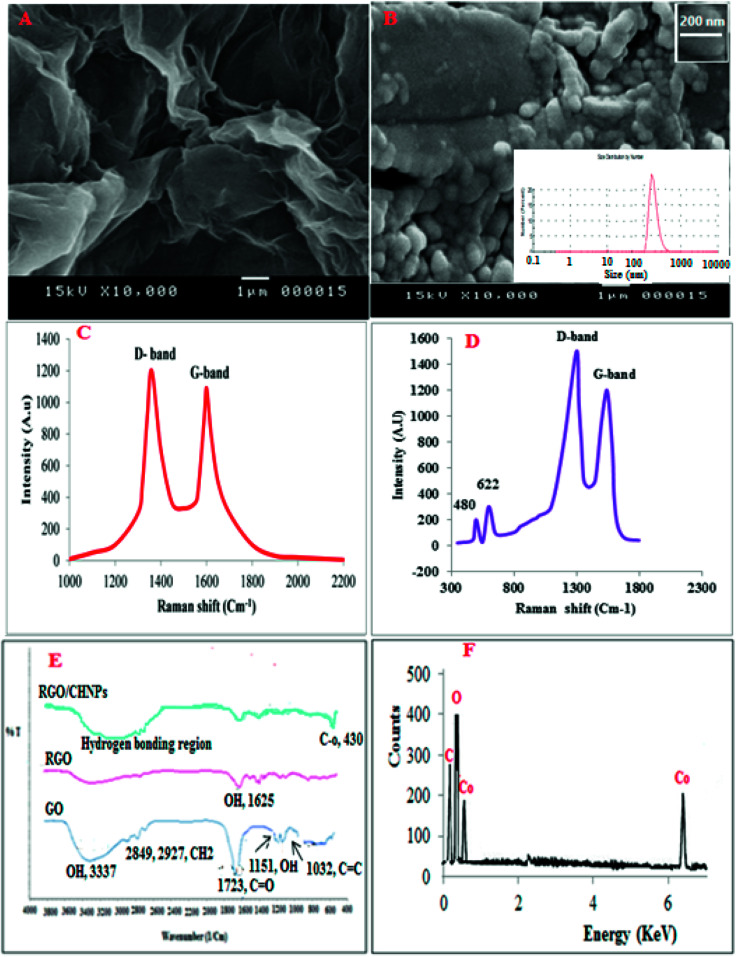
SEM of RGO (A) and RGO/CHNPs (B) with particle size distribution inset, Raman spectrum of RGO (C), Raman spectra of RGO and CHNPs (D), FTIR of GO, RGO and RGO/CHNPs (E) while (F) is EDX of RGO/CHNPs.

Suitable aliquot of VELP solution within calibration curve, 5.0 mL of PEG 6000 (5% v/v), 0.0035 g graphene nanoparticles, 0.0075 g of cobalt hydroxide nanoparticles adsorbent, 12 mL of Na_2_SO_4_ solution (0.05 mol L^−1^), and 5.0 mL of Britton–Robinson buffer solution (pH 7) were added to a 50 mL volumetric flask and diluted to the mark with double distilled water. This solution was transferred to a 50 mL centrifuge tube, heated at 75 °C in an ultrasonic bath for 25 min. and then cooled in an ice-bath immediately. The solution was then centrifuged for 10 min at 5000 rpm. After decantation, 2 mL of DMSO was added to the surfactant-rich phase and centrifuged at 5000 rpm for 10 min. to strip the analyte. This solution was taken and its fluorescence intensity was measured at 415 nm using the excitation wavelength of 350 nm. A blank solution was also treated similarly. Fig. 2S† illustrates the representative diagram for the proposed analytical method.

### 2.7. Real samples preparation

#### 2.7.1. Epclusa® tablets

The developed method was applied to different samples including Epclusa® tablets (100 mg velpatasvir + 400 mg sofosbuvir). The contents of ten tablets were accurately weighed, finely powdered, and thoroughly mixed in a mortar. Portions equivalent to the content of one tablet were accurately weighed and dissolved in a 20 mL methanol. The contents were sonicated for 20 minutes to ensure complete solubility. The filtrate was transferred to a 100 mL calibrated flask and diluted to the mark with methanol and subjected to the proposed procedure.

#### 2.7.2. Human blood plasma

Samples were collected from healthy volunteers at Assiut University Clinics three hours (*T*_max_ of the drug) after oral administration of the drug. Blood samples were collected from a convenient forearm vein into heparinized tubes, and then centrifuged at 6000 rpm for 15 min at room temperature, and then the plasma was separated and stored at −20 °C until analysis. For the determination of VELP in plasma samples, 1 mL of acetonitrile was added to 0.5 mL of the sample and shaken for 5 min for precipitation of proteins and prevents their interference. This solution was then centrifuged, filtered through a filter paper (Whatman no. 40), transferred to a 50 mL volumetric flask and subjected to the proposed procedure before and after spiking with the drug.

#### 2.7.3. Human urine

Urine samples were collected from healthy volunteers into glass tubes and stored in the refrigerator at 4 °C until analysis. The urine samples were filtered using a filter paper and 5 mL of the filtrate solution was treated using the recommended procedure before and after spiking with the drug.

The experimental protocol was conducted according to the Egyptian regulations and approved by the Institutional Human Ethics Committee, Assiut University, Assiut, Egypt. Informed consents were obtained from the human participants for this study.

## 3. Results and discussions

### 3.1. Rationale of the design and selection of sorbent

Graphene oxide (GO) was usually produced from oxidation of graphite flakes and have abundant OH and C=O functionalities. These groups have ability to form hydrogen bond networks with PEG 6000 based micelles hindering its elution from adsorbents. So, reduction of GO into RGO should be performed to prevent such cases of unwanted adsorption. Unfortunately, the process of reduction of GO into RGO is incomplete. So, another type of nanoparticles such as CHNPs should be added to achieve two main goals: (i) formation of hydrogen bonds with RGO and subsequently decrease binding affinity with the PEG 6000 based micelles and (ii) increasing surface area along with RGO for further selective binding of targeted molecules.^21–26^ In addition, RGO was used with CHNPs as a capping agent to prevent their aggregation.^27^ Moreover, the main advantages of CPE are separation and preconcentration of hydrophobic species.^24^ This technique was preferably used to enhance penetration of hydrophobic species into micelles so that the target analyte can be extracted even in presence of complexed matrices. Interestingly, the entrapment of the VELP within the micelle enhanced its fluorescence response due to further increase in rigidity of the drug.

RGO and CHNPs as adsorbents were used for preconcentration and separation of VELP from different matrices. They have two important properties that affect the adsorption efficiency: (a) they have a relatively high surface area and large number of active sites leading to high adsorption capacity and efficiency and (b) they are easily separated from solution even by using a very low amount of them.

For fluorometric detection, different excitation wavelengths were attempted in such a way that the highest emission intensity would be obtained. The maximum fluorescence intensity for VELP was observed at 415 nm upon excitation at 350 nm which was selected for intensity measurement. The emission wavelength was not shifted considerably after CPE. The fluorescence spectra of VELP after the CPE procedure using RGO and RGO/CHNPs were shown in Fig. 1. It was found that CHNPs has a great influence on fluorometric response of VELP. This may due to efficient precipitation process that have produced smaller particles size of CHNPs (200 nm) (Fig. 2B) increasing surface area along with RGO and could potentiate more intense hydrogen bonding which may decrease binding affinity with adsorbed micelles.

### 3.2. Characterization of RGO and CHNPs

Scanning electron microscopy (SEM) was used to characterize the RGO and CHNPs (Fig. 2A and B). The morphology of RGO and CHNPs shows a network-like structure which consists of interconnected nanoparticles. Interestingly, the nanoparticles possess a huge porous surface with numerous boundaries that can improve the adsorption efficiency of VELP for its quantitation in real samples. The SEM image shows the agglomerated CHNPs with an average size of 200 nm.

Raman spectroscopy was utilized to characterize RGO and RGO/CHNPs. From Fig. 2C and D, it was found that D-band the vibration of sp^2^-bonded carbon atoms appeared at 1346 cm^−1^. A G-band, the vibration of sp^3^-hybridized carbon, emerged at 1595 cm^−1^, which are typical Raman features of RGO. After addition of CHNPs, the development of two new bands at 480 and 622 cm^−1^ indicates further modification with CHNPs.

Further FTIR was used to characterize RGO where the bands obtained in Fig. 2E distinguished between graphene oxide (GO), reduced graphene oxide (RGO) and RGO–CHNPs combination. A broad band between 3500 cm^−1^ and 2500 cm^−1^ in the FTIIR spectrum of graphene oxide is due to OH stretching mode. The FTIR bands positioned at 2927 cm^−1^ and 2849 cm^−1^ are due to the asymmetric and symmetric CH_2_ stretching of graphene oxide, respectively while the band around 1720 is attributed to C=O stretches of carboxylic group. The band at 1619 cm^−1^is attributed to C=C stretching, 1224 cm^−1^ corresponds to C–OH stretch of alcohol group, 1080 cm^−1^ is attributed to C–O stretching vibrations of C–O–C.^28^ The reduction of graphene oxide was also characterized by FTIR spectroscopy. As shown in Fig. 2E, all the intensities of the bands corresponding to the oxygen containing functionalities of reduced graphene oxide were decreased as compared to the intensities of peaks of graphene oxide and even some were disappeared.

The FTIR spectrum in Fig. 4E provides information about interactions between the CHNPs. The FTIR band at 1625 cm^−1^ assigned to the stretching vibrations of OH groups and physically adsorbed H_2_O, respectively. The band at 435 cm^−1^ could be assigned to the Co–O stretching mode.

**Fig. 3:**
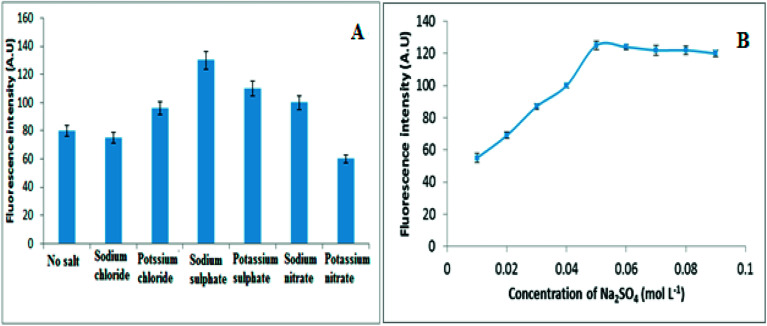
(A) The influence of different electrolytes and (B) Influence of Na_2_SO_4_ concentration on fluorescence intensity of 30 ng mL^−1^ of VELP.

**Fig. 4:**
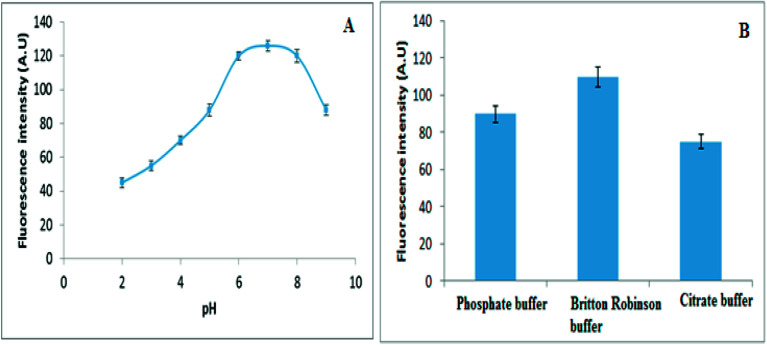
(A) The influence of pH and (B) Influence of different buffers on fluorescence intensity of 30 ng mL^−1^ of VELP.

EDX in Fig. 2F represents energy dispersive X-ray analysis of RGO-NPs/CHNPs nanocomposite which showed definite identifiable peaks of C, O and Co.

### 3.3. Optimization of variables

#### 3.3.1. Effect of type and concentration of the electrolytes

The salting-out of electrolytes such as KNO_3_, KCl, Na_2_SO_4_, K_2_SO_4_ and NaCl decrease the cloud point temperature and hence the separation of the surfactant-rich phase could be obtained at lower temperatures, which is very important for practical aspects.^28^ Therefore the effect of different salts such as K_2_SO_4_, KNO_3_ and NaCl as different electrolytes was investigated. The results presented in Fig. 3A have revealed that the fluorescence intensity was higher in the presence of Na_2_SO_4_. It was found from Hofmeister series that the highest salting out cations and anions are Na^+^ and SO_4_^2−^ and can be related to ions from Gibbs' free energy of hydration.^13^ Sulphate anions attract water molecules and strengthen hydrophobic interaction more than any other anion. Thus it was selected as a best electrolyte and its concentration was optimized. Moreover, the fluorescence intensity increases gradually with the Na_2_SO_4_ concentration up to 0.05 mol L^−1^ but beyond that, it becomes almost constant (Fig. 3B). This may be attributed to increasing the concentration of salt could increase the water viscosity accompanied by the surfactant rich phase, and hence disturb the phase separation. Therefore, the Na_2_SO_4_ concentration of 0.05 mol L^−1^ in the final solution was selected for further work.

#### 3.3.2. The effect of pH

The pH of the sample solution plays an important role in most analytical methods. The effect of pH on the fluorescence intensity of VELP after CPE in the range of 2–9 was studied. The pH of the solutions was adjusted to the desired value by the addition of 0.5 mol L^−1^ of HCl or NaOH. The results presented in Fig. 4A have indicated that the fluorescence intensity increased up to pH 6 and was constant in the range of 6–8. Based on the results pH 7 was selected as optimum. Upon increasing pH of the solution the solubility of VELP was considerably decreased^29^ that encouraged distribution of the drug to the hydrophobic core of micelles as a result the fluorescence intensity has increased with increasing pH value. With further increasing of pH, the free OH^−^ may occupy active sites of adsorbents and interlinking probability between adsorbed micelles and adsorbent and thus hindering its elution.

Moreover, different buffer systems such as citrate, phosphate and Britton–Robinson were investigated for maximum fluorescence intensity. The maximum signal intensity was observed in presence of Britton–Robinson buffer. Consequently the pH of the solution was adjusted by adding 5.0 mL of Britton–Robinson buffer (pH 7), Fig. 4B.

#### 3.3.3. Effect of surfactant type and concentration

Type and concentration of surfactant play an important role in enhancing fluorescence intensity of VELP after CPE. Two types of non-ionic surfactant with different molecular weights were chosen such as Triton X-100, Triton X-114, PEG 2000, PEG 4000 and PEG 6000 for investigating their suitability for this work. According to the obtained experimental results shown in Fig. 3S_A_,† PEG 6000 was chosen because of its extraction performance in producing a more viscous phase and also higher fluorescence intensity. Therefore the effect of surfactant amount on CPE was investigated to determine the minimum amount of surfactant which produces the maximum extraction efficiency. For this study different volumes of PEG (5% v/v) were added to the solution and the recommended procedure was carried out. Fig. 3S_B_† shows the effect of PEG 6000 volumes on the fluorescence intensity of VELP after CPE. The results indicate that the intensity increased up to 5 mL of the surfactant and remains constant for 5 and 6 mL. At higher volumes than 6 mL the surfactant rich phase volume increased and the coacervate phase is not viscous enough for phase separation. Thus 5 mL of PEG 6000 (5% v/v) was selected for easy phase separation and achieving the maximum analytical signal.

#### 3.3.4. The effect of incubation time and temperature

Carrying out CPE process at the lowest temperature and in the shortest equilibrium time will be highly desirable. So, the effects of the equilibrium temperature and time on the proposed CPE procedure for VELP determination were investigated in the ranges of 40–100 0C and 10–55 min, respectively. The results shown in Fig. 4S† have indicated that the highest analytical signal is achieved when the solution is incubated at 75 hC for 25 min. The need for this short time may be attributed to high surface area between adsorbent and sample solution that could facilitate the rapid mass transfer of analyte from aqueous phase to the adsorbent surface. Really, sonication can also help to decrease the contact time of the analyte and the sorbent to the lowest possible time.

#### 3.3.5. Effect of RGO and CHNPs amounts as an efficient adsorbents

**Fig. 5:**
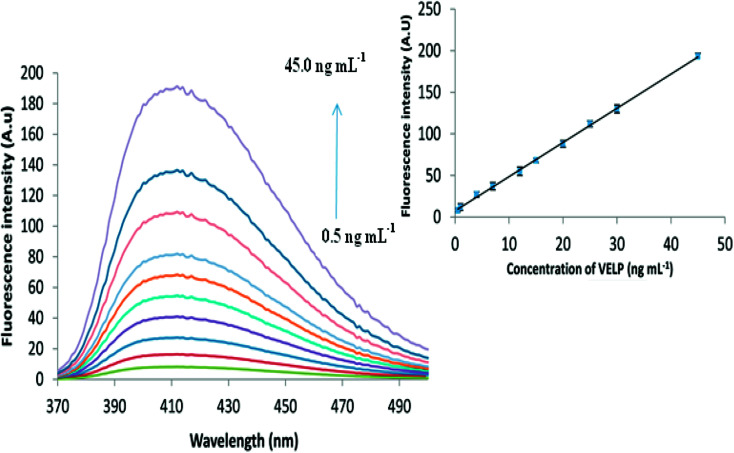
The fluorescence spectra for different concentrations of VELP after CPE and the corresponding calibration curve in the range of 0.5–45.0 ng mL^−1^.

The effect of RGO and CHNPs amounts as sorbents was studied in the range of 0.0015–0.005 and 0.005–0.009 g, respectively. The results obtained revealed that the signal intensity was increased to a maximum value upon using 0.0035 g of RGO and 0.0075 g of CHNPs. So, these amounts of sorbents were used as optimized amounts for fluorometric detection of VELP in its real samples, Fig. 5S_A_ and 5S_B_.† From these figures, it was found that increase amounts of RGO and CHNPs lead to constant fluorescence intensities. So, 0.0035 g of RGO and 0.0075 g of CHNPs were used as ideal adsorbents amounts.

#### 3.3.6. Effect of stripping solvent

Different volumes of ethanol, acetonitrile, DMSO and DMF were used as a stripping solvent to preconcentrate VELP from its bulk solution. It was found that maximum fluorescence intensity was obtained with 2 mL of DMSO.

## 4. Method validation

The method was validated in accordance with ICH guidelines for evaluation of various parameters that include linearity, precision, accuracy, limit of detection, limit of quantitation and selectivity.^30^

### 4.1. Linearity and calibration curves

From Fig. 5, it is obvious that increase in the fluorescence intensity is observed as the concentration of VELP is increased in the range of 0.5 to 45 ng mL^−1^ under optimum conditions. The inset of Fig. 5 shows that there is a good correlation between the fluorescence intensity and concentration of the drug. Calibration parameters are given in Table 1. In case of biological samples, the obtained calibration curves were 1.2–30.0 and 1.5–42.0 ng mL^−1^ for spiked plasma and urine, respectively.

**Table 1:** Quantitative parameters for the analysis of VELP by the proposed method and comparison with other reported methods

Method	Linearity range (ng mL^−1^)	LOQ (ng mL^−1^)	LOD (ng mL^−1^)	Reference
RP-HPLC	25–150 × 10^3^	8.3 × 10^3^	2.74 × 10^3^	7
	5–25 × 10^3^	2.4 × 10^3^	0.8 × 10^3^	8
	1–60 × 10^3^	0.003 × 10^3^	0.001 × 10^3^	9
Spectrofluorometry	0.5–45.0	0.12	0.04	This work

### 4.2. Limit of detection (LOD) and quantitation (LOQ)

The limits of detection (LOD) and quantification (LOQ) were calculated based on the standard deviation (SD) of the intercept and the slope of the linear calibration curve (Table 1). The LOD was expressed as 3.3 *σ*/*S*, while LOQ was expressed as 10 *σ*/*S*, where (*σ*) is the standard deviation of the intercept and (*S*) is the sensitivity parameter expressed by the slope of the calibration curve. The LOD and LOQ for VELP were calculated for six replicates and were found to be 0.04 and 0.121 ng mL^−1^, respectively. The performance of the proposed method was compared with the reported RP-HPLC methods [7–9], Table 1. Moreover, the LOQ was found to be 1.2 and 1.5 ng mL^−1^ for the drug in plasma and urine, respectively.

### 4.3. Inter-day and intra-day precision

Intra-day and inter-day precision were expressed in terms of RSD as recommended by the ICH guidelines. The results especially % RSD given in Table 1S† have proved the precision of the proposed fluorometric method. The good reproducibility of the method indicated by % RSD may be attributed to the efficient adsorption of unique nanoparticles.

### 4.4. Accuracy

To prove the accuracy of the proposed method, the results obtained from VELP analysis were compared with those obtained by the reported method^7^ both in pure form and in tablets. Statistical analysis of the results obtained by the proposed and reported methods using Student's *t*-test and variance ratio F-test showed no significant difference between them regarding accuracy and precision, respectively, as shown in Table 2.

**Table 2:** Determination of VELP in pure form and tablets using proposed and reference methods

Form	Concentration a	Mean % recovery ± SD b		*t*-value c	*F*-value c
		Proposed method	Reference method^7^		
Pure	30	99.7 ± 1.14	98.5 ± 1.23	1.85	1.12
	40	98.9 ± 1.55	100.23 ± 1.65	1.76	1.33
Epclusa® tablets	30	97.84 ± 1.35	98.02 ± 1.44	1.22	1.11
	40	98.13 ± 1.54	98.56 ± 1.36	1.64	1.02

a Concentration was calculated in ng mL^−1^ and μg mL^−1^ for proposed and reference method, respectively.

b Average of five replicates.

c Theoretical values at 95% confidence limit: *t* = 2.31, *F* = 6.39.

Moreover, the accuracy of the proposed procedure was checked by applying the standard addition method. The results obtained from the standard addition method were satisfactory as indicated in Table 2S.† The obtained recovery ranged from 98.2 ± 1.8–101.5 ± 1.8 (%), which indicates high accuracy of the proposed method.

Intra-day precision, inter-day precision and accuracy in biological samples were determined by analyzing QC samples at three concentration. Accuracy and precision data for intra- and inter-day human plasma and urine samples are presented in Table 3. The results for all samples (intra- and inter-day) were found to be within the acceptance criteria or method validation.^31^

**Table 3:** Accuracy and precision of the proposed method for the analysis of VELP in spiked human plasma and urine

Urine samples					Plasma samples				
Inter-day assay (*n* = 5)		Intra-day assay (*n* = 5)		Concentration (ng mL^−1^)	Inter-day assay (*n* = 5)		Intra-day assay (*n* = 5)		Concentration (ng mL^−1^)
Precision (%RSD)	Accuracy (%)	Precision (%RSD)	Accuracy (%)		Precision (%RSD)	Accuracy (%)	Precision (%RSD)	Accuracy (%)	
1.18	98.6	1.59	97.1	5	1.57	97.2	1.67	99.2	3
1.16	100.7	1.62	99.4	10	1.35	99.6	1.34	99.3	10
1.75	98.1	1.64	101.5	30	1.45	99.6	1.32	98.5	20

### 4.5. Interference study

The tolerance limit was defined as the maximum concentration of potential interfering substances which caused ±5% error in the measurement of VELP. To investigate the selectivity of the proposed method the effect of different compounds on the determination of 10 ng mL^−1^ of VELP was examined (Table 4).

**Table 4:** Effect of interfering species on the determination of 10 ng mL^−1^ VELP

Interfering species (X)	Tolerance ratios (X)/VELP
**I-Common excipients**	
Glucose	5000
Lactose	4000

**II-Biological active compounds**	
Ascorbic acid	10 000
Uric acid	7000
Dopamine	8000

**III-Co-administered drugs**	
Sofosbuvir	1200
Omeprazole	5000
Pantoprazole	5000
Ribavirin	4000

**IV-Common cations and anions**	
Ca^2+^, Mg^2+^	15 000
Cu^2+^, Zn^2+^	14 000
Na^+^, K^+^	20 000
C_2_O_4_^2-^	15 000
SO_4_^2−^, CO_3_^2-^	12 000

### 4.6. Robustness

To study the robustness of the proposed method, slight but deliberate changes were made in some parameters, such as pH, RGO amount, volume of surfactant, CHNPs amount, incubation time and temperature. It was found from Table 3S† that slight variation not affect significantly on recovery percentage.

### 4.7. Application to real samples

To evaluate the applicability of the proposed method, tablets containing the cited drug were determined by standard addition method without interference from common excipients.

Moreover, the plasma and urine samples were analyzed by the proposed method after 3 hours of storage and after spiking with different concentration of VELP. Acetonitrile was used for precipitation of plasma proteins while hydrophobicity based CPE was sufficient to remove interference from urine soluble proteins.

The results in Table 5 could show that good recoveries were obtained for the samples. Therefore, the method can be used as an efficient quantitative method for studying the pharmacokinetics or pharmacodynamics of VELP.

**Table 5:** Determination of VELP in plasma and urine samples by the proposed method

Sample	Standard added (ng mL^−1^)	Standard found (ng mL^−1^) (±SD^a^)	% Recovery
Plasma	—	14.45 ± 0.21^b^	—
	5	19.24 ± 0.32	98.92
	10	23.95 ± 0.42	97.96
Urine	5	5.15 ± 0.09	103.00
	10	10.05 ± 0.18	100.50

a Average of five replicates.

b Analyzed after 3 hours of collection.

## 5. Reusability of adsorbent

The reusability and regeneration of RGO–CHNPs adsorbent was studied. This was performed by reusing the adsorbent for extraction of VELP at the first adsorption–desorption cycle to the six cycle where the fluorescence intensities were 125.45 and 119.65, respectively. From these, we can deduce that the adsorbent can be reused at least six times without remarkable change in its adsorption capacity.

## 6. Conclusion

This study presents a useful combination of dispersive solid phase extraction (dSPE) and cloud point extraction (CPE) for spectrofluorometric detection of VELP for the first time. The dSPE and CPE were based on RGO/CHNPs sorbent and PGE 6000 nonionic surfactant, respectively. The dSPE approach was very useful for separation and preconcentration of target analyte while CPE has removed the impurities and eliminated the possible interference of other species with VELP in tablets, plasma and urine samples. The developed procedure exploiting highly affordable and simple experimental setup provides an extremely simple, inexpensive, efficient, environmentally friendly and ultrasensitive approach with satisfactory precision (repeatability and reproducibility) and accuracy for determination of VELP.

## Conflicts of interest

Authors declare no conflict of interest.

## Supplementary Material

RA-008-C7RA13719B-s001
