# Supplementary material for: Enhanced dispersive solid phase extraction assisted by cloud point strategy prior to fluorometric determination of anti-hepatitis C drug velpatasvir in pharmaceutical tablets and body fluids
Source: RSC Adv. 2018 Apr 10;8(24):13292–300. doi: 10.1039/c7ra13719b (PMC9079717; doi:10.1039/c7ra13719b)
Supplement: RA-008-C7RA13719B-s001 [file RA-008-C7RA13719B-s001.pdf]

Electronic Supplementary Material (ESI) for RSC Advances.
This journal is © The Royal Society of Chemistry 2018

Supplementary information

Enhanced solid phase extraction synergistically assisted by cloud point strategy prior to the determination of a recently FDA approved anti-hepatitis C drug velpatasvir: Application in Biological Fluids

Mohamed M. El-Wekil[*1], Hassan Refat H. Ali[1], Adel A. Marzouk[2], Ramadan Ali[3]

[1] Department of Pharmaceutical Analytical Chemistry, Faculty of Pharmacy, Assiut University, Assiut, Egypt.

[2] Department of Pharmaceutical Chemistry, Faculty of Pharmacy, Al Azhar University, Assiut, Egypt.

[3] Department of Pharmaceutical Analytical Chemistry, Faculty of Pharmacy, Al Azhar University, Assiut, Egypt.

* Corresponding author: mohamed.mohamoud@ymail.com

Figures

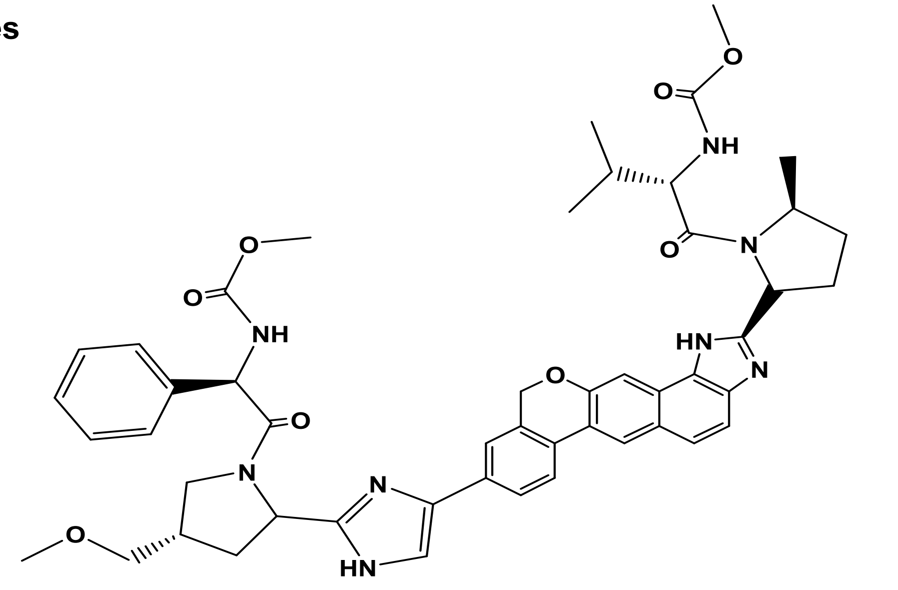

Fig.1S: The chemical structure of VELP.

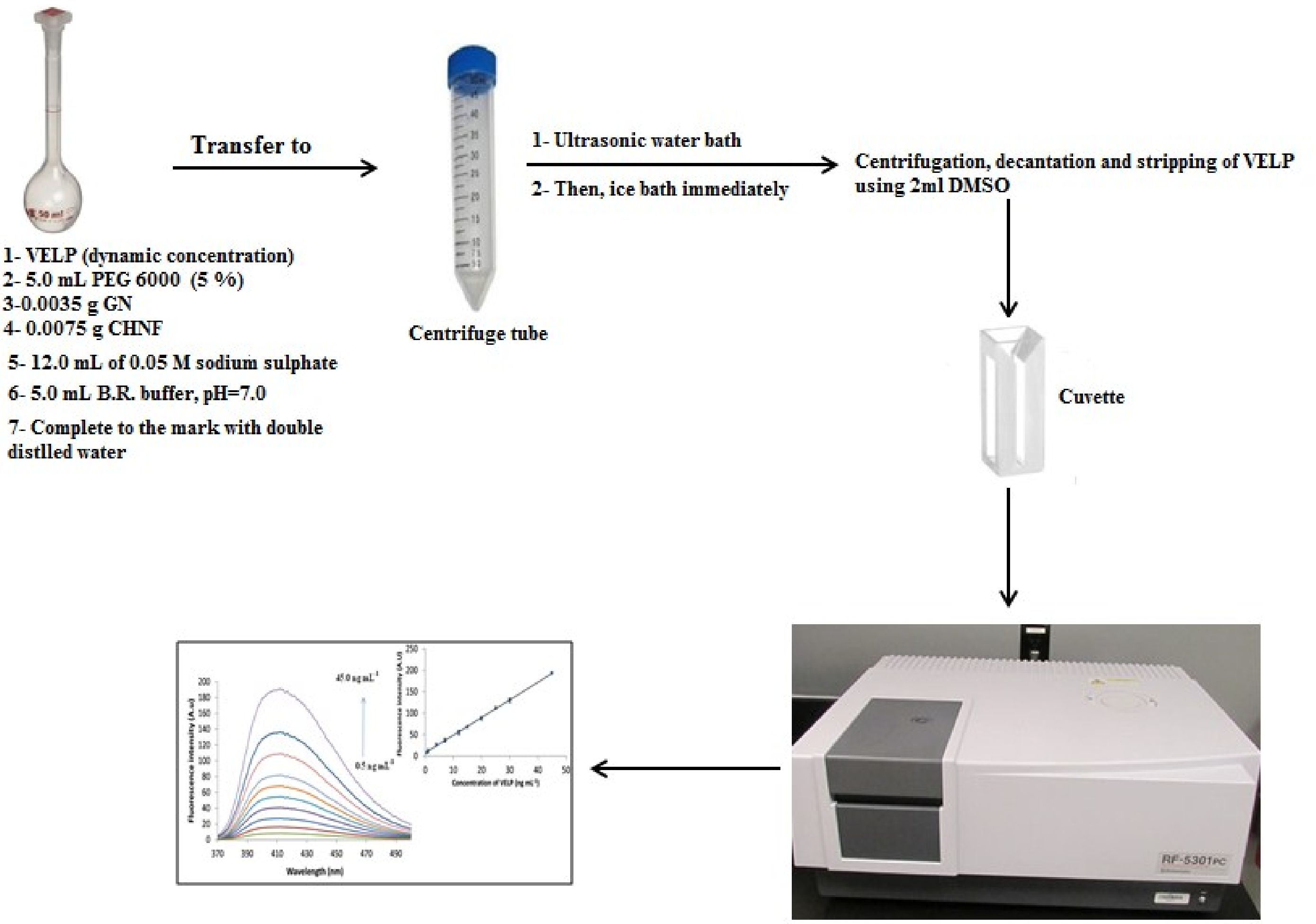

Fig.2S: Graphical representation of the proposed analytical procedure.

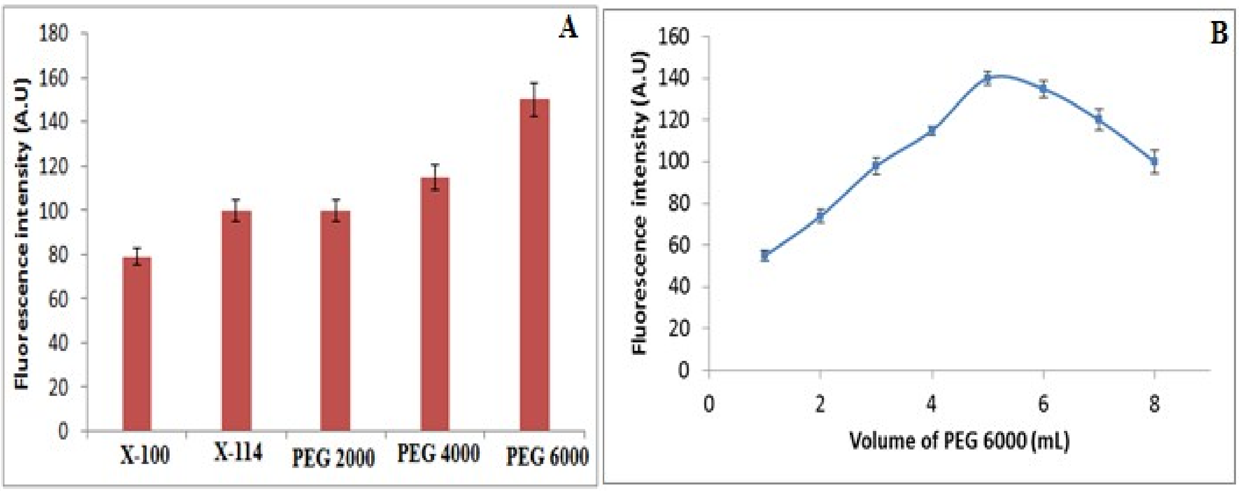

Fig. 3S: (A) The influence of surfactant type and (B) Influence of different volumes of PGE 6000 on fluorescence intensity of 30 ng/ml of VELP.

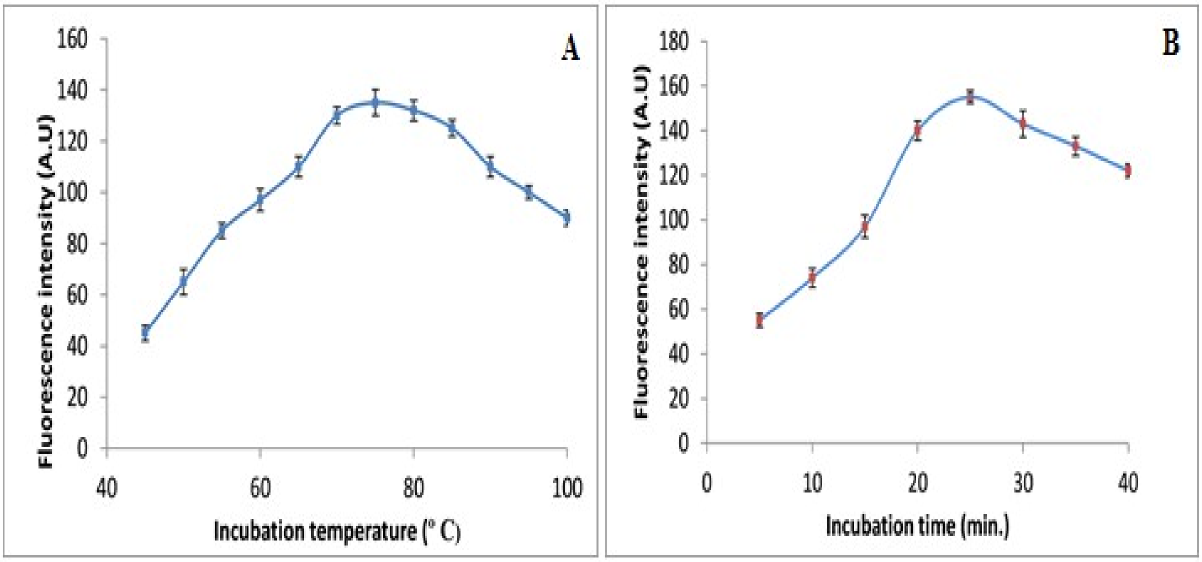

Fig. 4S: (A) The influence of incubation temperature and (B) Influence of incubation time on fluorescence intensity of 30 ng/ml of VELP.

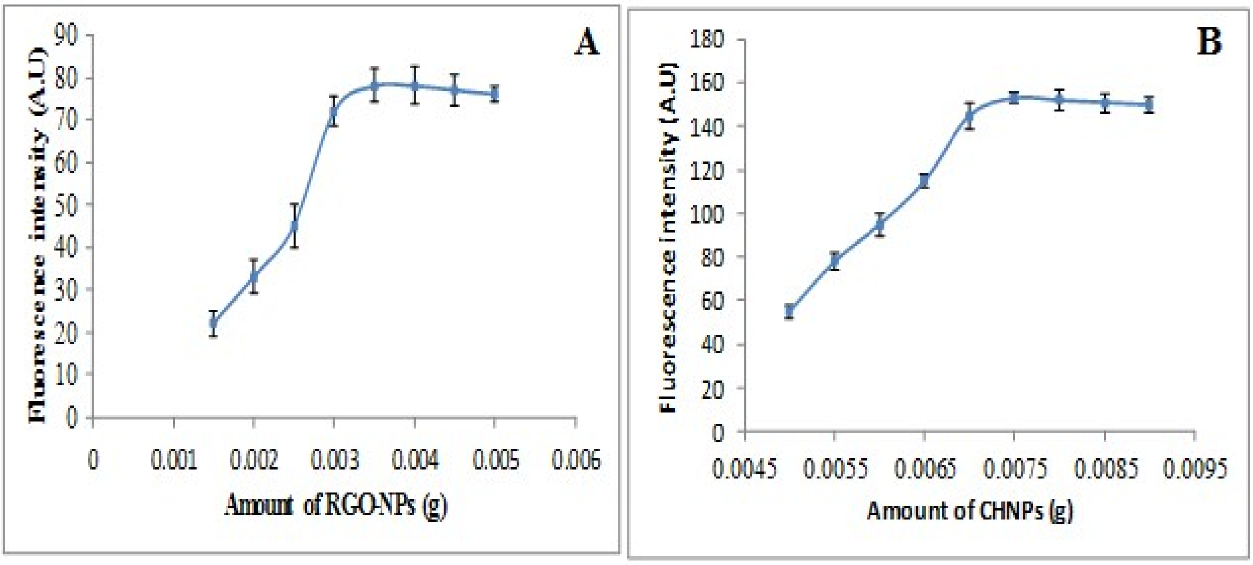

Fig. 5S: (A) The influence of RGO-NPs amount using 0.0075 g CHNPs while (B) The influence of CHNPs amount using 0.0035 g of RGO-NPs on fluorescence intensity of 30 ng/ml of VELP.

Tables

Table 1S. The precision of the proposed method for determination of VELP.

Concentration of VELP (ng mL^{-1})	Inter-day precision		Intra-day precision	
	% Recovery± SD*	% RSD	% Recovery± SD*	% RSD
10	99.5±1.8	1.81	100.9±2.6	2.57
20	100.3±2.5	2.49	97.8± 1.7	1.73
30	98.8±1.9	1.92	102.3±2.0	1.96

* Average of five replicates.

Table 2S. Recovery study for determination of VELP in tablets by standard addition method.

Amount taken ($ng\ ml^{-1}$)	Amount of standard added ($ng\ mL^{-1}$)	Amount found ($ng\ mL^{-1}$)	% Recovery ± SD*	% RSD
	2	1.98	99.0± 1.7	1.72
	5	4.98	99.6±2.2	2.20
5	10	10.15	101.5± 1.8	1.77
	15	15.08	100.5±2.4	2.38
	20	19.88	99.4± 1.5	1.51
	25	24.55	98.2± 1.8	1.83

* Average of five replicates.

Table 3S. The robustness of the proposed method.

Variables	% Recovery (±SD*)	RSD
No variation	99.7± 2.1	2.11
pH		
6.0	98.2± 2.0	2.04
8.0	98.7± 2.3	2.33
Volume of surfactant (mL)		
4.5	99.7± 2.1	2.11
5.5	99.5± 1.8	1.81
Amount of RGO-NPs (g)		
0.003	100.2±2.3	2.29
0.004	99.8±1.7	1.70
Amount of CHNPs (g)		
0.007	97.6±2.5	2.56
0.008	97.2±1.9	1.95
Incubation temperature (ºC)		
70	100.8±2.4	2.38
80	97.5±2.7	2.77
Incubation time (min.)		
23	99.7±2.6	2.61
27	101.3±2.0	1.97

* Average of six replicates.
